# Oxymatrine Ameliorates Memory Impairment in Diabetic Rats by Regulating Oxidative Stress and Apoptosis: Involvement of NOX2/NOX4

**DOI:** 10.1155/2020/3912173

**Published:** 2020-11-16

**Authors:** Yongpan Huang, Xinliang Li, Xi Zhang, Jiayu Tang

**Affiliations:** ^1^Department of Clinic, Medicine School, Changsha Social Work College, Changsha, Hunan, China; ^2^Institute of Chinese Medicine, Hunan Academy of Chinese Medicine, Changsha, China; ^3^Department of Neurology, Brain Hospital of Hunan Province, Changsha, China

## Abstract

Oxymatrine (OMT) is the major quinolizidine alkaloid extracted from the root of *Sophora flavescens Ait* and has been shown to exhibit a diverse range of pharmacological properties. The aim of the present study was to investigate the role of OMT in diabetic brain injury *in vivo* and *in vitro*. Diabetic rats were induced by intraperitoneal injection of a single dose of 65 mg/kg streptozotocin (STZ) and fed a high-fat and high-cholesterol diet. Memory function was assessed using a Morris water maze test. A SH-SY5Y cell injury model was induced by incubation with glucose (30 mM/l) to simulate damage *in vitro*. The serum fasting blood glucose, insulin, serum S100B, malondialdehyde (MDA), and superoxide dismutase (SOD) levels were analyzed using commercial kits. Morphological changes were observed using Nissl staining and electron microscopy. Cell apoptosis was assessed using Hoechst staining and TUNEL staining. NADPH oxidase (NOX) and caspase-3 activities were determined. The effects of NOX2 and NOX4 knockdown were assessed using small interfering RNA. The expression levels of NOX1, NOX2, and NOX4 were detected using reverse transcription-quantitative PCR and western blotting, and the levels of caspase-3 were detected using western blotting. The diabetic rats exhibited significantly increased plasma glucose, insulin, reactive oxygen species (ROS), S-100B, and MDA levels and decreased SOD levels. Memory function was determined by assessing the percentage of time spent in the target quadrant, the number of times the platform was crossed, escape latency, and mean path length and was found to be significantly reduced in the diabetic rats. Hyperglycemia resulted in notable brain injury, including histological changes and apoptosis in the cortex and hippocampus. The expression levels of NOX2 and NOX4 were significantly upregulated at the protein and mRNA levels, and NOX1 expression was not altered in the diabetic rats. NOX and caspase-3 activities were increased, and caspase-3 expression was upregulated in the brain tissue of diabetic rats. OMT treatment dose-dependently reversed behavioral, biochemical, and molecular changes in the diabetic rats. *In vitro*, high glucose resulted in increases in reactive oxygen species (ROS), MDA levels, apoptosis, and the expressions of NOX2, NOX4, and caspase-3. siRNA-mediated knockdown of NOX2 and NOX4 decreased NOX2 and NOX4 expression levels, respectively, and reduced ROS levels and apoptosis. The results of the present study suggest that OMT alleviates diabetes-associated cognitive decline, oxidative stress, and apoptosis via NOX2 and NOX4 inhibition.

## 1. Introduction

Diabetes mellitus (DM) is a common metabolic disorder associated with chronic complications, including nephropathy, angiopathy, retinopathy, and peripheral neuropathy [1]. It is widely accepted that diabetes can lead to cognitive impairment to some extent [2]. An increasing amount of evidence, including experimental and clinical studies, has demonstrated the alterations in cognitive deterioration, in particular cognitive dysfunction as a result of diabetes [3]. Oxidative stress has emerged as a critical pathogenic factor involved in the initiation and development of diabetic complications [4]. Diabetes is accompanied by overproduction of reactive oxygen species (ROS) in various organs and tissues, including the brain, retina, kidney, and heart [5]. ROS production in diabetes is predominantly mediated by various NADPH oxidases (NOXs). NOXs are a major source of ROS in nonphagocytic cells and are critical mediators in brains afflicted by the diabetic milieu. The NOX family of proteins contains seven members, including NOX1-5, DUOX1, and DUOX2 [6]. Among these, NOX2 and NOX4 have been shown to be the primary sources of ROS in multiple types of brain disease, including ischemic stroke and traumatic brain injury [7–9]. Increasing evidence has now demonstrated that NOXs serve a variety of essential roles requiring deliberate ROS generation in the brain [10]. The deleterious role of NOX-derived ROS in the pathogenesis of diabetes may result in the initiation of neuronal damage [11, 12]. However, the mechanisms underlying NOX-mediated neuronal dysfunction in diabetic brain injury remain largely unknown and still require further study.

Oxymatrine (OMT) is a major component extracted from *Sophora flavescens Ait*. OMT possesses multiple pharmacological properties, including anti-inflammatory [13], antiviral [14], antioxidative, antifibrotic [15, 16], and antiapoptotic activity [14, 17]. To date, the majority of studies have focused on the anti-inflammatory and antifibrotic properties of OMT, which have been shown to be mediated by modulation of the NF-*κ*B, JAK/STAT, and TLR4 signaling pathways. Recently, numerous studies showed the neuroprotective effects of OMT against diabetes. Our previous study demonstrated that endothelial dysfunction following diabetes was due to upregulated expression of NOX4 in the thoracic aorta, which resulted in increased oxidative stress in diabetes [18]. Thus, it was hypothesized that OMT inhibited diabetic brain injury through regulation of NOX2/NOX4. To test this hypothesis, a rat model of diabetes and an *in vitro* high-glucose model were used. The aim of the present study was to assess the neuroprotective effects of OMT on diabetic brain injury via inhibition of NOX2/NOX4 activation *in vitro* and *in vivo*.

## 2. Materials and Methods

### 2.1. Animal Experiments

Male Sprague-Dawley rats weighing 250-300 g were purchased from Hunan SJA Laboratory Animal Co., Ltd. (Xiang 2018-00A1). Animals were housed in the following conditions: 12 h light/dark cycle, at 22-25°C and 50-65% humidity. All animals were given *ad libitum* access to food and water. The study was performed in accordance with the guidelines described in the *Guide for the Care and Use of Laboratory Animals* (NIH Publication No. 85-23, revised 1996), and the experiments were approved by the Hunan Academy of Chinese Medicine Animal Care and Use Committee.

### 2.2. Induction and Assessment of Diabetes

To simulate type 2 diabetes, first, all the rats were fed a high-fat and high-cholesterol diet. After 4 weeks, diabetes was induced in the rats by intraperitoneal injection of a single dose of 65 mg/kg streptozotocin (STZ) that was freshly dissolved in citrate buffer (pH 4.4, 0.1 mol/l). Control rats were treated with citrate buffer only. A total of 48 h after STZ injection, blood samples were collected and plasma glucose levels were measured using an enzymatic glucose oxidase peroxidase diagnostic kit. Rats with fasting plasma glucose levels > 250 mg/dl were defined as diabetic and used for further experiments. Animals in each experiment were randomly assigned to one of six groups (*n* = 10 per group): (i) control group, normal rats that received a saline injection intraperitoneally (physiological saline 0.1 ml/100 g); (ii) model diabetes mellitus (DM) group, diabetic rats that received a saline injection intraperitoneally (saline 0.1 ml/100 g); DM+OMT groups (iii) 30, (iv) 60, and (v) 120 mg/kg, and (vi) a vehicle group (DM) (*n* = 10). The OMT dosage and dosing frequency were selected based on previous studies [15, 17]. The rats were intragastrically administered saline or OMT for 7 weeks and then intraperitoneally injected with normal saline.

After 10 weeks, learning and memory functions were evaluated for 5 consecutive days in a Morris water maze.

### 2.3. Morris Water Maze Test

The Morris water maze test was performed as described previously [19, 20]. The Morris water maze pool was 160 cm in diameter, 50 cm high, and 29 cm deep. The platform had a diameter of 12 cm and a height of 27 cm and was fixed at 2 cm below the water surface of the southeast quadrant (target quadrant). The camera lens was placed at the center of the pool, 2 cm from the bottom of the pool to record the movements of the rat. The water temperature was maintained at 22 ± 1°C. The experiments lasted for 5 days, including the hidden platform tests and space exploration experiments. On the first 4 days, the hidden platform tests were performed, and on the last day, space exploration experiments were performed.

The pool was placed in the center of a large room containing various visual cues and divided into four equal quadrants, north (N), south (S), east (E), and west (W). The cues remained constant throughout the study. A translucent acrylic platform was submerged ~1 cm below the water surface (for the navigation test) or removed from the tank (for the spatial probe test). The water maze task was used for 5 consecutive days.

### 2.4. Learning Test

The experiments lasted for 4 days. During the test, the platform position was fixed in the target quadrant (SE quadrant), and the water was inserted into the water from the four quadrants. During the training, the animals were gently placed in the water and the amount of time it took for the animals to find the platform was recorded. Once the animals had found the platform, they were left on for 10 sec. If the platform was not found within 90 sec, the time was recorded as 90 sec and the animal was placed on the platform for 10 sec. At the end of the training, the rats were returned to the cage and kept warm. Rats were trained at each of the four water intake points once a day, and for the final experimental trials, an average of four repeats was used. The order of water entry per animal per day was consistent, but the order of different animals in the same cage was different to exclude the possibility of exchange of animal information within the group.

### 2.5. Measurement of Blood Glucose and Serum Insulin Levels

After fasting the rats for 12 h overnight, they were anesthetized and sacrificed. Blood samples were collected from the abdominal aorta, allowed to clot for 30 min at 4°C, and centrifuged (3,500 × g, 10 min, 4°C), and the supernatant was used for measurement of glucose and insulin. Blood glucose was estimated using a commercially available glucose kit based on the glucose oxidase method. Insulin was measured by a radioimmunoassay method [18].

### 2.6. Determination of S-100B Levels in Plasma

Commercially available ELISA kits (BioSource) were used to determine the levels of S-100B proteins in plasma/brain homogenates. Analyses of all samples, standards, and controls were run in duplicate, according to the manufacturer's protocol.

### 2.7. Nissl Staining

Nissl staining was performed using a 0.1% cresyl violet solution (Sigma-Aldrich; Merck KGaA) using a standard protocol to evaluate the injury of diabetic rats [18]. In total, 6 coronal sections between the anterior edge and posterior edge of the brain were collected and processed for Nissl staining. The peri-impact area was defined as the region in the cortex. Every sixth coronal tissue section was chosen at random to quantify cell survival. Brain sections were examined using a light microscope (×200). Neuronal survival in the brain was quantified using ImageJ (National Institutes of Health).

### 2.8. Cell Culture

SH-SY5Y cells were obtained from American Type Culture Collection. The SH-SY5Y cells were authenticated by Short Tandem Repeat identification. SH-SY5Y cells were cultured in DMEM supplemented with 10% FBS, 100 m/ml penicillin, and 100 m/ml streptomycin and maintained in a humidified atmosphere of 5% CO_2_ at 37°C. The medium was changed every 2 days. Cells were incubated with OMT (8 *μ*m/l) for 1 h, after which, cells were treated with glucose (30 mmol/l) for 48 h. All assays consisting of appropriate controls were performed in triplicate and repeated on three separately initiated cultures.

### 2.9. Measurement of ROS

The total ROS level was measured using a reactive oxygen species assay kit. According to the manufacturer's protocol, intracellular ROS levels were determined by measuring the oxidative conversion of cell permeable 2V,7V-dichlorofluorescein diacetate (DCFH-DA) to fluorescent dichlorofluorescein (DCF) using a fluorospectrophotometer (F4000). Briefly, tissues or cells were incubated with control media or 10 Ag/ml LPC for 4 h in the absence or presence of DPI (100 or 300 AM) or allopurinol (100 AM) followed by washing with D-Hank's and incubating with DCFHDA at 37°C for 20 min. Then, DCF fluorescence distribution was detected using fluorospectrophotometer analysis at an excitation wavelength of 488 nm and at an emission wavelength of 535 nm.

### 2.10. Determination of Apoptosis Using Hoechst 33258 Staining

For detection of apoptosis, Hoechst 33258 staining was performed as previously described [21, 22]. After treatment with glucose, SH-SY5Y cells were fixed with fixing solution for 5 min. Subsequently, SH-SY5Y cells were washed with PBS three times and incubated in Hoechst 33258 solution (0.5 ml) for 5 min, then washed with PBS three times. Apoptosis of SH-SY5Y cells was examined under a fluorescence microscope.

### 2.11. Small Interference (si)RNA Transfection

The SH-SY5Y cells were inoculated in 6-well plates and transfected with 20 nM control siRNA or validated NOX2 siRNA (cat. no. sc-35503) or NOX4 siRNA (cat. no. sc-36149) from Santa Cruz Biotechnology, Inc., using RPMI 1640 media. Briefly, 5 *μ*l NOX2 and NOX4 siRNA (20 *μ*M) were mixed with RPMI 1640 media. Separately, Lipofectamine 2000 (Invitrogen; Thermo Fisher Scientific, Inc.) was mixed with RPMI 1640 media, and the mixtures were then combined for 20 min at room temperature. The Lipofectamine-siRNA mixture was then added to each well containing cells and medium and incubated for 6 h. The medium was subsequently replaced with antibiotic-free ECM supplemented with 5% FBS for 24 h. The cells were harvested 48 h after transfection.

### 2.12. Determination of Apoptosis in Brain Tissue

Tissue apoptosis was determined using TUNEL staining. Briefly, brain tissues were excised and fixed in 4% paraformaldehyde in PBS at room temperature for 24 h. Fixed tissues were embedded in paraffin and stained using a TUNEL kit (Roche Diagnostics). The apoptotic index (percentage of TUNEL-positive nuclei/total number of nuclei) was determined.

### 2.13. Ultrastructural Studies

Electron transmission microscopic examination was performed as previously described [23]. Hippocampal tissues (diameter = 1 mm) were fixed in 4% glutaraldehyde for 24 h, rinsed with 0.1 M phosphate buffer, fixed with 2% acid, dehydrated with acetone, soaked with epoxy resin embedding agent, pure acetone, embedded into a block, and cut into a semithin section (2 *μ*m). Ultrathin sections were stained with uranyl acetate and lead bismuth citrate and observed using a Zeiss Libra 120 transmission electron microscope (Carl Zeiss AG).

### 2.14. Caspase-3 Activity Assay

Caspase-3 activity was measured according to the manufacturer's protocol (Beyotime Institute of Biotechnology). Briefly, 10 *μ*l brain tissue homogenates or SH-SY5Y cell lysates were mixed with 90 *μ*l reaction solution containing caspase-3 substrate (Ac-DEVD-pNA) and incubated at 37°C for 60 min. The absorbance at 450 nm was recorded. Enzyme activity is presented as U/g protein and 1 U of enzyme and is defined as the amount of enzyme required to cleave 1.0 nmol AcDEVD-pNA per hour at 37°C.

### 2.15. Determination of NOX Activity

NOX activity was determined as previously described [24, 25]. The assay was based on detecting the reduction of ferrocytochrome c by the superoxide anion formed by NOX in the presence of NADPH. NOX activity was evaluated after exposure of brain tissue and SH-SY5Y cell homogenates to GD for 15, 30, and 60 min. The homogenates were incubated with SDS (100 *μ*M) and ferrocytochrome c (100 *μ*M; Sigma-Aldrich; Merck KGaA) for 5 min at 37°C. The reaction was initiated by the addition of NADPH (250 *μ*M), and the reduction of ferrocytochrome c was measured spectrophotometrically at 550 nm for 5 min. In order to test the specificity of the reaction for NOX and to discard an effect of XaO or cPLA2 inhibitors on NOX activity, cells exposed to GD for 30 min were homogenized and enzyme activity was measured in the presence of apocynin, AEBSF, allopurinol, or AACOCF3, which were added to the reaction buffer. Results are expressed as nmol/h/mg protein.

### 2.16. Reverse Transcription-Quantitative (RT-q)PCR

Gene expression levels (mRNA) in cartilage were determined using RT-qPCR. Total RNA was extracted (Takara, China) and purified with 75% ethanol, and its concentration was determined by spectrophotometry. The purified total RNA (200 ng per sample) was added to a transcription kit (cat. no. DRR037A; Takara Bio, Inc.) and mixed to generate the first strand template (reverse transcription reaction). The sequences of the primers used for qPCR were the following: NOX1 forward, 5′-ATA TTT TGG AAT TGC AGA TGA ACA-3′ and reverse, 5′-ATA TTG AGG AAG AGA CGG TAG-3′; NOX2 forward, 5′-GGA GAA TTA ACC CCT GCC A-3′ and reverse, 5′-GGC TAG CTG GAG AAG ACC AC-3′; NOX4 forward, 5′-TGT TGG ATG ACT GGA AAC CA-3′ and reverse, 5′-TGG GTC CAC AAC AGA AAA CA-3′; and GADPH forward, 5′-CAA CAG CCT CAA GAT CAT CAG CA-3′ and reverse, 5′-TGG CAT GGT CTG TCA TGA GT-3′. qPCR was performed using a real-time PCR system (ABI 7300). Expression levels were determined quantitatively using SYBR Premix ExTaq (Takara Bio, Inc.).

### 2.17. Western Blot Analysis

For western blotting analysis, 40 *μ*g protein per sample was loaded on a 10% gel and resolved using SDS-PAGE. The proteins were transferred to a PVDF membrane; the membranes were blocked using 5% milk and subsequently incubated with rabbit anti-NOX2, NOX4, full-length caspase-3, cleaved caspase-3, and *β*-actin antibodies (Santa Cruz Biotechnology, Inc.) at 4°C overnight. Subsequently, the membrane was washed and incubated with secondary antibodies (horseradish peroxidase-conjugated) at 4°C, and the protein bands were visualized using an enhanced chemiluminescence kit (Amersham Biosciences). Finally, the expression levels of protein were calculated using a Molecular Imager ChemiDoc XRS system (Bio-Rad Laboratories, Inc.). *β*-Actin was used as the loading control.

### 2.18. Statistical Analysis

SPSS version 21.0 was used for statistical analysis. Data are presented as the mean ± standarddeviation or standard error of the mean. The differences between groups of data were analyzed using a one-way ANOVA. *P* < 0.05 was considered to indicate a statistically significant difference.

## 3. Results

### 3.1. OMT Improves Blood Glucose and Insulin Levels and Diabetes-Induced Cognitive Deficits in Diabetic Rats

**Figure 1:**
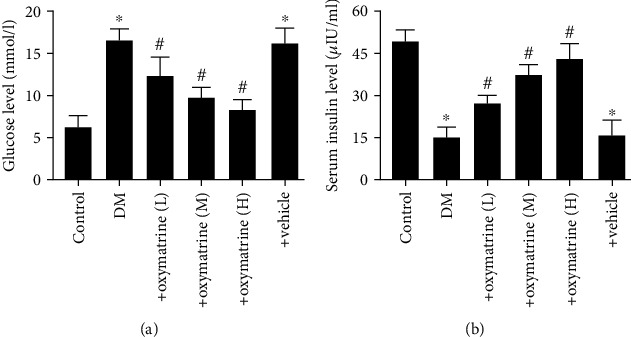
OMT improves blood glucose and insulin levels in diabetic rats. (a) Blood glucose and (b) serum insulin levels. Data are presented as the mean ± standarddeviation. ^∗^*P* < 0.05 vs. control; ^#^*P* < 0.05 vs. DM. DM: diabetes mellitus; DM+oxymatrine (L): DM+ 30 mg/kg OMT; DM+oxymatrine (M): DM+OMT 60 mg/kg; DM+oxymatrine (H): DM+oxymatrine+120 mg/kg OMT. OMT: oxymatrine.

To determine whether STZ injection-induced diabetes and to assess the protective effects of OMT against diabetic brain injury, blood glucose and insulin levels were assessed. A diabetic rat model was constructed, and the rats exhibited higher fasting blood glucose levels compared with the control. As shown in Figure 1(a), diabetic rats had a blood glucose level > 11.1 mmol/l, which was significantly higher compared with the control group. OMT significantly decreased the blood glucose levels of diabetic rats in a dose-dependent manner. Plasma insulin levels were not significantly altered by OMT treatment (Figure 1(b)). These results suggest that OMT treatment may be a potential therapeutic strategy for the treatment of DM.

To assess the effects of OMT on the cognitive function in diabetic rats, a Morris water maze test (on the 10^th^ week) was used. After 12 weeks of diabetes induction, the diabetic rats exhibited notable cognitive impairment. The mean escape latency for the trained animals was reduced from 60 sec to 20 sec over the course of the 20 learning trials. There were no significant differences between the groups on the first day of testing in the Morris water maze. On the second day, the transfer latency was notably different longer in diabetic rats (52.21 ± 1.35 sec) and the control group (32.15 ± 1.27 sec) (*P* < 0.05). OMT (30, 60, and 120 mg/kg) dose-dependently decreased the mean escape latency (*P* < 0.05).

**Figure 2:**
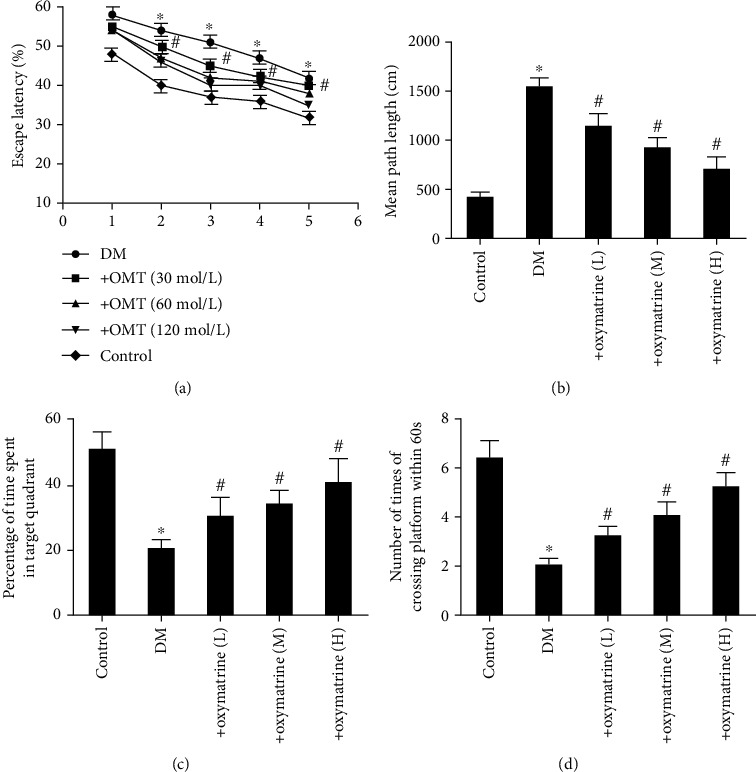
Effects of OMT on the cognitive function in diabetic rats. (a) Mean percentage of time spent in the target quadrant, (b) mean path length, and (c) spatial memory acquisition phase in control and diabetic rats. (d) Number of platform crosses. *n* = 10. Data are presented as the mean ± standarddeviation. ^∗^*P* < 0.05 vs. control; ^#^*P* < 0.05 vs. DM. DM: diabetes mellitus; OMT: oxymatrine.

As shown in Figure 2(a), diabetic rats spent more time finding the platform and learning its location during the training session. OMT reversed the poorer performance in a concentration-dependent manner, as indicated by the decrease in latency from the 2^nd^ training day (*P* < 0.05). The results of mean path length, shown in Figure 2(b), also demonstrated a significant increase in the diabetic rats for 4 consecutive days of training (*P* < 0.05).

To investigate how the animals learned and consolidated the platform location during the experiments, the probe trial was assessed. The results first exhibited a significant difference (*P* < 0.05) during the 4^th^ training day. As shown in Figure 2(c), there was a decline in the time spent in the target quadrant in diabetic rats compared with the control rats (*P* < 0.05). Compared with the diabetic rats, as indicated in Figure 2(d), OMT increased the time spent in the target quadrant significantly (*P* < 0.05) and decreased the number of times the animals crossed the former platform location (*P* < 0.05). OMT improved this index significantly in diabetic rats (*P* < 0.05).

### 3.2. OMT Decreases Oxidative Stress Injury in the Brain Tissues of Diabetic Rats

**Figure 3:**
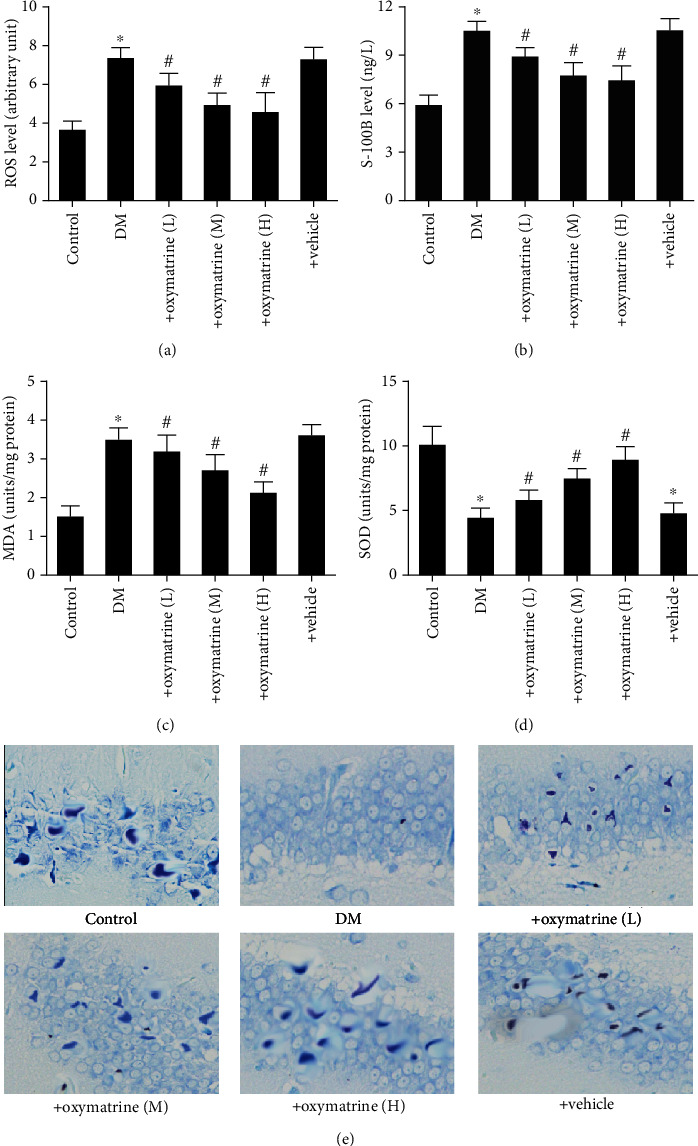
OMT decreases oxidative stress injury of brain hippocampal tissue in diabetic rats. (a) ROS, (b) S-100B, (c) SOD, and (d) MDA levels. (e) Nissl staining. Data are presented as the mean ± thestandarderrorofthemean. ^∗^*P* < 0.05 vs. control; ^#^*P* < 0.05, ^#~^*P* < 0.001 vs. DM. DM: diabetes mellitus; OMT: oxymatrine; ROS: reactive oxygen species; MDA: malondialdehyde; SOD: superoxide dismutase.

Oxidative stress is an important cause of brain injury. To investigate whether OMT exhibited protective effects on oxidative stress-related brain injury in diabetic rats, the total ROS levels were determined. As shown in Figure 3(a), the ROS levels in the brain tissues were significantly increased in the diabetic rats compared with the control group, and OMT dose-dependently decreased the ROS levels of brain tissues.

Nissl staining showed that the neurons exhibited notable morphological alterations in diabetic rats compared with the control and OMT dose-dependently protected diabetic rats from morphological alterations of the Nissl body (Figure 3(b)). Consistently, the plasma S-100B levels were significantly increased in diabetic rats; OMT significantly decreased the plasma S-100B levels in a dose-dependent manner (Figure 3(c)). These results suggest that OMT ameliorated oxidative stress in diabetic rats.

Furthermore, the antioxidative index including SOD and MDA levels was assessed. The results showed that the activity of SOD decreased whereas MDA content increased. Additionally, OMT reversed these effects (Figures 3(d) and 3(e)).

### 3.3. OMT Ameliorates Oxidative Stress-Related Apoptosis in Diabetic Rats

**Figure 4:**
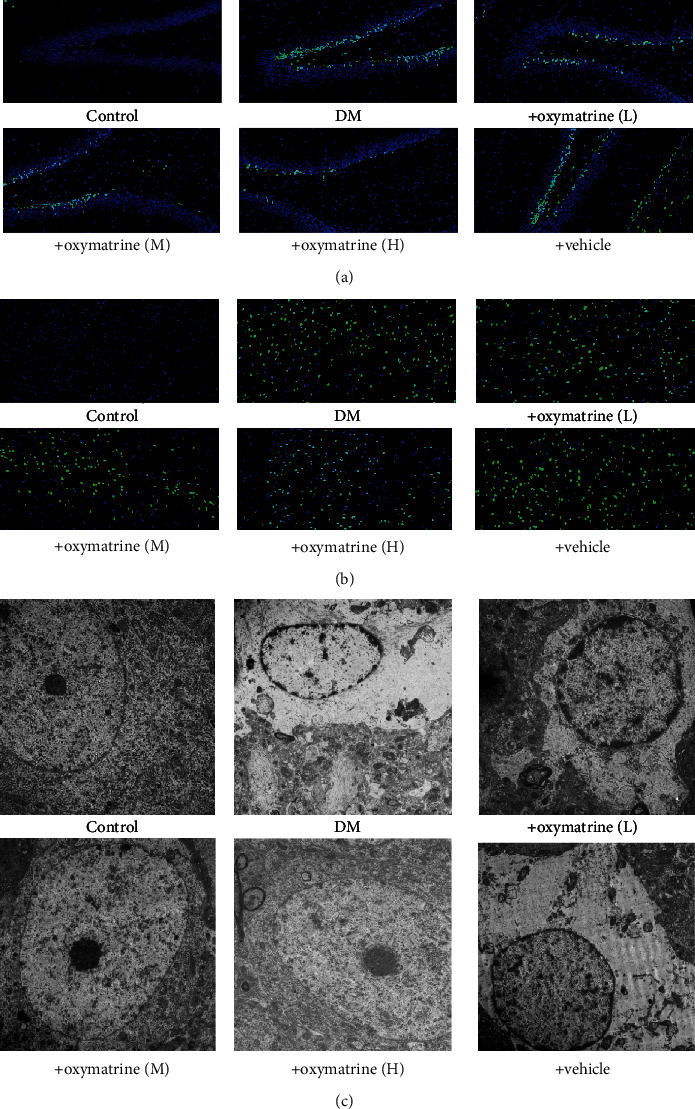
Effects of OMT on the apoptosis of brain tissue in diabetic rats. (a) TUNEL staining of hippocampal tissue. (b) TUNEL staining in the cortex. Magnification, ×200. (c) Electron micrograph of hippocampal tissue. OMT: oxymatrine.

As shown in Figures 4(a) and 4(b), there were no TUNEL-positive cells in the control group. Induction of diabetes significantly increased the number of TUNEL-positive cells, and this increase was significantly reduced by OMT. The vehicle did not show any effects on apoptosis. Electron microscopy was used to investigate the protective effects of OMT on ultrastructural changes. As shown in Figure 4(c), serious heterogeneous subcellular and extracellular space abnormalities were observed in the hippocampal tissues, which were attenuated by OMT.

### 3.4. OMT Decreases the Expression of NOX in Brain Tissues of Diabetic Rats

**Figure 5:**
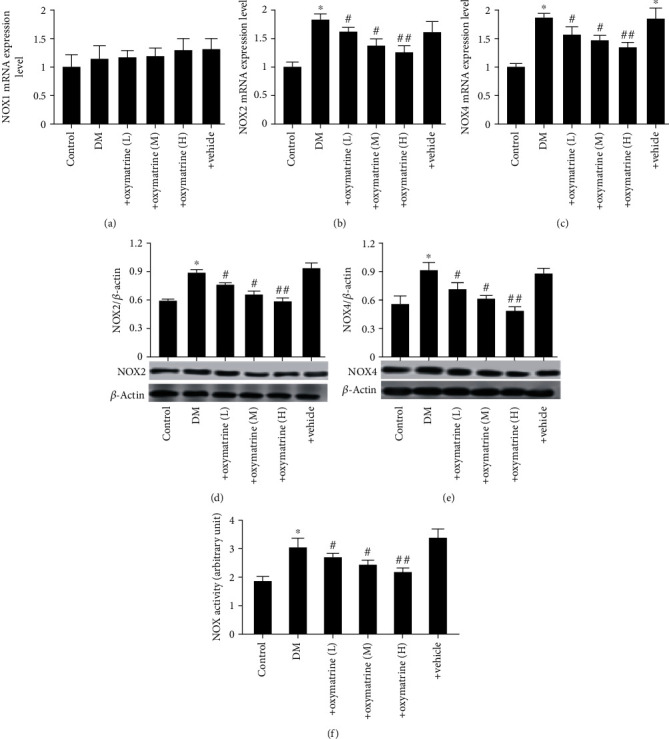
Effects of OMT on the expression of NOX in the brain tissues of diabetic rats. (a) NOX1, (b) NOX2, and (c) NOX4 mRNA expression levels. (d) NOX2 and (e) NOX4 protein expression levels. (f) NOX activity. Data are presented as the mean ± standarddeviation. ^∗^*P* < 0.05 vs. control; ^##^*P* < 0.01 vs. DM. DM: diabetes mellitus; OMT: oxymatrine; NOX: NADPH oxidase.

Whether OMT mediated protection against protected diabetes-related brain injury associated with NOX expression was next determined. As shown in Figure 5(a), there were no significant changes in NOX1 expression in the diabetic rat brains compared with the control group, whereas diabetes significantly elevated NOX2 and NOX4 expression levels in the brain tissues compared with the control group. OMT significantly decreased NOX2 and NOX4 expression levels in the brain tissues of type 2 diabetes rats in a dose-dependent manner (Figures 5(b)–5(e)). Consistently, total NOX enzyme activity was significantly increased in brain tissues, and OMT treatment dose-dependently decreased the total NOX enzyme activity of hippocampal tissues (Figure 5(f)). These results suggest that OMT attenuated diabetic brain injury through the downregulation of NOX2 and NOX4 expression, but not NOX1.

### 3.5. OMT Decreases Oxidative Stress and Apoptosis of SH-SY5Y Cells Treated with High Glucose

**Figure 6:**
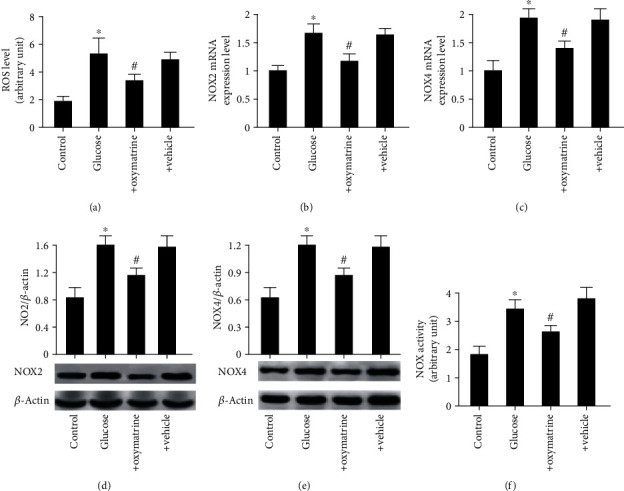
OMT decreases oxidative stress in SH-SY5Y cells treated with high glucose: (a) ROS levels; (b) NOX2 and (c) NOX4 mRNA expression levels; (d) NOX2 and (e) NOX4 protein expression levels; (f) NOX activity. Data are presented as the mean ± standarddeviation. ^∗^*P* < 0.05 vs. control; ^#^*P* < 0.05, ^##^*P* < 0.01 vs. glucose. Glucose: 30 mM/l glucose; glucose+oxymatrine: 30 mM/l glucose+8 *μ*M OMT; glucose+vehicle: glucose+DMSO. OMT: oxymatrine; NOX: NADPH oxidase.

**Figure 7:**
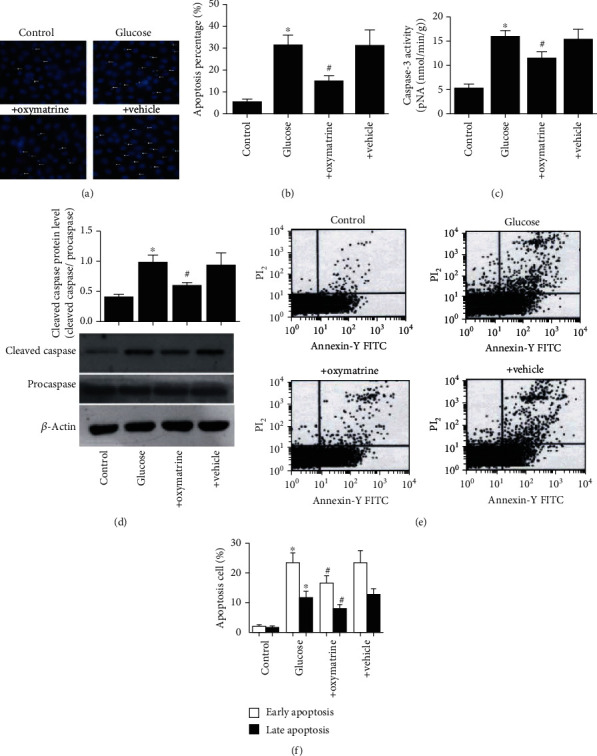
OMT inhibits oxidative stress and apoptosis in SH-SY5Y cells treated with high glucose. (a) Representative images of Hoechst staining. (b) Percentage of apoptotic cells. (c) Caspase-3 activity. (d) Caspase-3 protein expression levels. (e) Flow cytometry analysis of the annexin V-FITC/PI staining. (f) Quantification of annexin V-FITC/PI staining, showing the percentage of early (annexin V-FITC positive and PI negative) and late (annexin V-FITC positive and PI positive) apoptotic cells. Data are presented as the mean ± standarddeviation. ^∗^*P* < 0.05 vs. control; ^#^*P* < 0.05 vs. glucose. OMT: oxymatrine.

To further verify the results regarding OMT using an *in vitro* model of diabetes, a nerve cell (SH-SY5Y) model of high glucose was utilized to mimic the *in vivo* conditions. As shown in Figure 6(a), ROS levels were significantly increased in the SH-SY5Y cells treated with high glucose compared with the control group, and the increase in ROS levels was abolished by OMT. Consistent with the animal results, the expression levels of NOX2 and NOX4 and the total NOX enzyme activity were significantly increased in SH-SY5Y cells treated with high glucose. OMT significantly decreased NOX2 and NOX4 expression levels and the total NOX enzyme activity in SH-SY5Y cells treated with high glucose (Figures 6(b)–6(f)). Additionally, apoptosis was significantly increased in SH-SY5Y cells treated with high glucose compared with the control group, whereas OMT treatment significantly reduced this (Figures 7(a) and 7(b)). Similarly, the expression levels of cleaved caspase-3 and caspase-3 enzyme activity were significantly increased in SH-SY5Y cells treated with high glucose. The results showed increases in the proportion of apoptotic and necrotic cells in cells treated with high glucose compared with the control group. These alterations were reversed by OMT treatment in a dose-dependent manner (Figures 7(c)–7(f)).

### 3.6. Knockdown of NOX2 and NOX4 Decreases the Oxidative Stress and Apoptosis of SH-SY5Y Cells Treated with High Glucose

**Figure 8:**
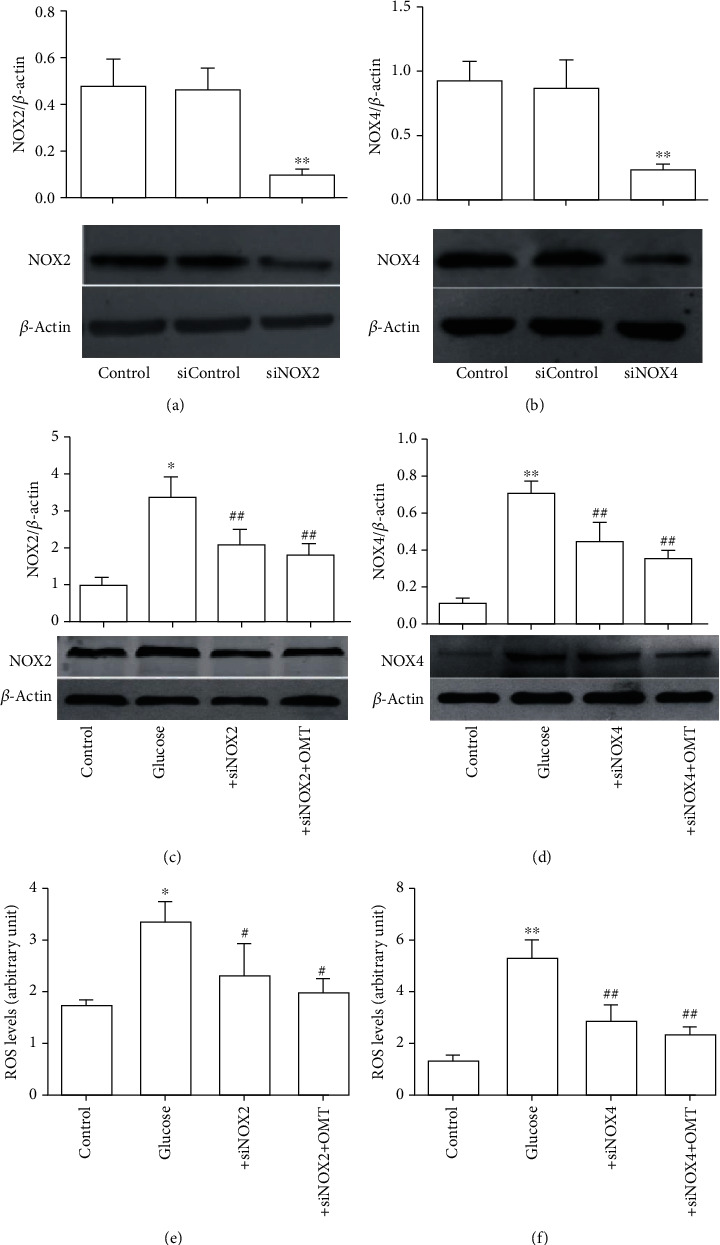
Knockdown of NOX2 and NOX4 reduced oxidative stress and apoptosis in SH-SY5Y cells treated with high glucose. (a, b) NOX2 and NOX4 siRNA transfection effectively reduced NOX2 and NOX4 expression levels. (c, d) NOX2 and NOX4 siRNA transfection effectively downregulated NOX2 and NOX4 expression levels in SH-SY5Y cells treated with high glucose and OMT. (e, f) NOX2 and NOX4 siRNA transfection effectively decreased ROS levels in SH-SY5Y cells treated with high glucose and OMT. Data are presented as the mean ± standarddeviation. ^∗^*P* < 0.05, ^∗∗^*P* < 0.01 vs. control; ^#^*P* < 0.05, ^##^*P* < 0.01 vs. glucose. OMT: oxymatrine; NOX: NADPH oxidase; ROS: reactive oxygen species; siRNA: small interfering RNA.

**Figure 9:**
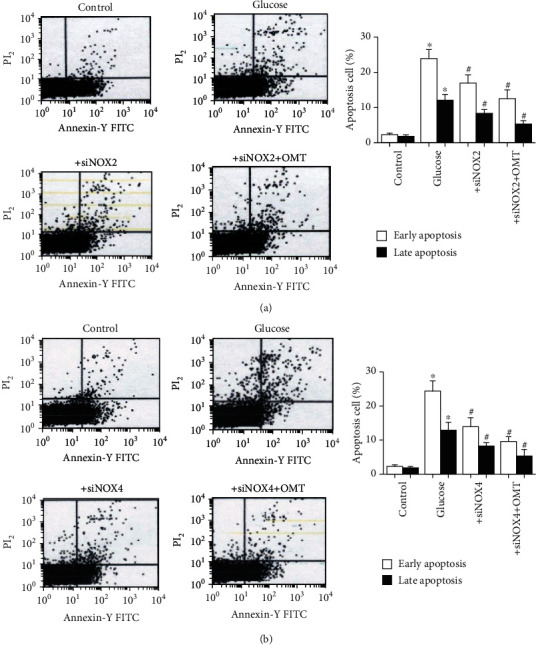
Knockdown of NOX2 and NOX4 reduced apoptosis in SH-SY5Y cells treated with high glucose. Flow cytometry analysis and quantitative analysis of annexin V-FITC/PI staining showing the percentages of early (annexin V-FITC positive and PI negative) and late (annexin V-FITC positive and PI positive) apoptotic cells. (a) NOX2 siRNA transfection effectively reduced apoptosis. (b) NOX2 siRNA transfection effectively reduced apoptosis. Data are presented as the mean ± standarddeviation. ^∗^*P* < 0.05 vs. control; ^#^*P* < 0.05 vs. glucose. NOX: NADPH oxidase.

To further assess the contributions of NOX2 and NOX4 in the high-glucose conditions, NOX2 and NOX4 expression was knocked down using NOX2- and NOX4-specific siRNAs. The knockdown efficiency shown in Figures 8(a)–8(d) showed that NOX2 and NOX4 knockdown was successful. In addition, knockdown of NOX2 and NOX4 significantly reduced the overproduction of ROS when cells were subjected to high-glucose conditions (Figures 8(e) and 8(f)). Furthermore, flow cytometry analysis showed that NOX2 and NOX4 knockdown reduced high-glucose-induced apoptosis (Figures 9(a) and 9(b)). Taken together, these findings suggest that OMT ameliorates oxidative stress and apoptosis through the inhibition of NOX2 and NOX4 activity.

## 4. Discussion

The major novel finding of the present study was that diabetic brain injury response culminates in the production of ROS through NOX enzymes and the linking of OMT to the inhibition of NOX activity and reduction in caspase-3 levels. In the present study, using a rat model of diabetes, generated by feeding rats with high-fat and high-cholesterol diets and intraperitoneal injection of STZ, or a nerve cell model of high glucose, the results showed that OMT significantly reduced blood glucose and insulin levels, improved cognitive dysfunction and histological changes, inhibited oxidative stress, and suppressed neuronal apoptosis in diabetic rats and SH-SY5Y cells. Moreover, OMT downregulated the expression of NOX2, NOX4, and caspase-3 and decreased NOX and caspase-3 activity. These results suggest that OMT ameliorates diabetes-induced injuries related to inhibition of NOX2, NOX4, and apoptosis *in vivo* and *in vitro*.

There is growing evidence showing the long-term effects of diabetes on the brain and that it manifests at a structural, neurophysiological, and neuropsychological level, and multiple pathogenic factors appear to be involved in the pathogenesis of cerebral dysfunction in diabetes, such as episodes of hypoglycemia, cerebrovascular alterations, the role of insulin in the brain, and the involvement of hyperglycemia-induced impairments [26–28]. One of the causes of cerebral dysfunction in the diabetic brain relates to oxidative stress mediated by free radicals. Oxidative stress, one of the primary contributors to cerebral pathogenesis, accelerates brain aging and tissue damage in diabetes mellitus [29, 30]. A recent study showed that oxidative stress and changes in the antioxidative defense systems impaired learning and memory in rats. Wang and Jia [19] showed that decreased antioxidant status was an important mechanism underlying cognitive impairment in diabetic rats. Oxidative stress has been shown to cause diabetes-induced aortic endothelial dysfunction via NOX4 activation [31]. In addition, NOX4 activation, which serves as the predominant source of O_2_· production, is observed in diabetes-induced vascular dysfunction. Therefore, NOX activation may be considered an important mechanism underlying increased oxidative stress in diabetes [32]. Studies have shown that NOX2 and NOX4 expression and activity were significantly upregulated under several conditions, including brain ischemia/reperfusion injury [33], hypertension [34], hypoxia-related diseases [35], and DOX-induced cardiomyopathy [36]. Based on these studies, it is reasonable to speculate that NOX-derived ROS accumulation may be responsible for the impairment of SH-SY5Y cell function when treated with high glucose. The present study showed that NOX2 and NOX4 expression and activity were significantly increased in SH-SY5Y cells treated with high glucose, accompanied by an increased NOX-derived ROS production as well as deteriorated function of cells. Knockdown of NOX2 and NOX4 partially inhibited the increase in oxidative stress and NOX2 and NOX4 expression and activity in SH-SY5Y cells treated with high glucose. Furthermore, numerous studies have demonstrated that NOX2 and NOX4 activation underlies the perturbation in cellular homeostasis in diabetes [37, 38]. It is well known that excessive production of ROS under diabetic circumstances contributes to reduced antioxidant capacity, leading to diabetic injuries, including neuropathy, nephropathy, vascular dysfunction, and retinopathy, and initiating mitochondrial oxidative damage, which accelerates cell apoptosis and/or necrosis. Studies in experimental models and in patients have demonstrated that oxidative stress is the primary contributor to diabetes-related complications [39, 40]. Several studies have confirmed that STZ-induced diabetic rats exhibit high levels of MDA, combined with increased glutathione disulfide reductase activity, a lower GSH/GSSG ratio, and lower ATP levels [41, 42]. These studies highlight the involvement of oxidative stress in diabetes-associated cerebral disturbances. In the present study, the results showed that oxidative stress not only accelerated the pathogenesis of cerebral dysfunction but also significantly increased the ROS levels and S-100B levels in diabetes. Furthermore, the levels of SOD and MDA were shown to be upregulated in the brain tissues of diabetic rats. These results support our hypothesis. In the *in vivo* and *in vitro* experiments, OMT suppressed the ROS levels and NOX2 and NOX4 expression. These results suggest that attenuation of diabetes-associated cerebral injury by OMT is associated with its antioxidant properties.

A common theory attempting to explain the pathogenesis of cerebral dysfunction in diabetes relates to neuronal cell death caused by oxidative stress, which is mediated by oxygen free radicals. The brain is particularly vulnerable to oxidative damage as a result of high oxygen consumption rates, abundant lipid content, and a lack of antioxidant enzymes. Neuronal cells are sensitive to oxidative insults. Numerous studies have demonstrated that accumulation of ROS in diabetes is a critical factor underlying brain damage [42, 43]. Oxidative stress is an important contributor to diabetic complications and exacerbates cellular damages. Excessive production of ROS contributes to the initiation of programmed cell death in diabetes, which increased neuronal apoptosis in diabetes [44, 45]. In view of the important role of oxidative stress, strategies for intervention of ROS-induced cell apoptosis may be of particular relevance. As the primary active ingredient of *Sophora flavescens*, OMT has a wide range of pharmacological properties, including treatment of cardiovascular and cerebrovascular diseases. Our previous study confirmed that OMT inhibited autophagy-activated cell apoptosis/death via a MIF/mTOR signaling pathway [46]. In the present study, OMT was shown to ameliorate diabetes-related complications; specifically, the significant increases in neuronal apoptosis and caspase activity in the cerebral cortex and hippocampus were attenuated by treatment with OMT.

A Morris water maze test is a widely accepted behavioral test used for evaluating brain function related to spatial learning and memory. The present study evaluated cognitive function of the *in viv*o diabetes rat model. The results showed that the diabetic rats exhibited learning and memory deficits, consistent with previous studies [19, 27]. These learning and memory deficits were prevented by OMT treatment, suggesting that the ameliorative effects of OMT on the learning and memory deficits are associated with decreased apoptosis in diabetic rats.

In conclusion, the present study demonstrated that OMT exhibits beneficial effects by lowering blood glucose levels, reducing learning and memory deficits, suppressing oxidative stress, and decreasing caspase-3 expression, possibly through inhibition of NOX2 and NOX4 activation *in vivo* and *in vitro*. These novel findings may provide a pharmacological foundation for the use of OMT as treatment in diabetic patients.

## Data Availability

The datasets used and/or analyzed during the current study are available from the corresponding author on reasonable request.

## Supplementary Materials

Supplementary Materials The figure in the supplementary file represents the consequence of OMT on memory impairment.
