# Supplementary figures and images for: Oxymatrine Ameliorates Memory Impairment in Diabetic Rats by Regulating Oxidative Stress and Apoptosis: Involvement of NOX2/NOX4
Source: Oxid Med Cell Longev. 2020 Nov 16;2020:3912173. doi: 10.1155/2020/3912173 (PMC7683156; doi:10.1155/2020/3912173)

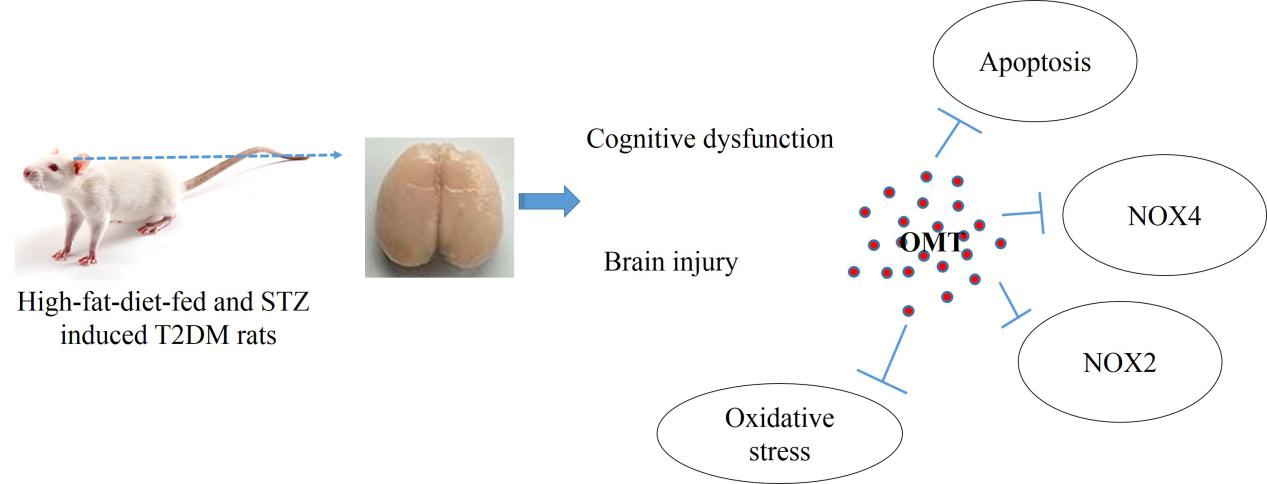


The consequence of OMT on memory impairment

Supplement: Supplementary Materials — The figure in the supplementary file represents the consequence of OMT on memory impairment. [file 3912173.f1.docx]
